# WOMEN AT RISK: SOCIOECONOMIC STATUS, LIFESTYLE FACTORS, AND BRAIN VULNERABILITY AMONG JAPANESE AND SWEDISH FEMALE

**DOI:** 10.1093/geroni/igac059.2909

**Published:** 2022-12-20

**Authors:** Yingxu Liu, Yasuko Tatewaki, Benjamin Thyreau, Yinghao Liu, Ye Zhang, Nina Karalija, Carl-johan Boraxbekk, Yasuyuki Taki

**Affiliations:** Institute of Development, Aging and Cancer, Tohoku University, Sendai, Miyagi, Japan; Institute of Development, Aging and Cancer, Tohoku University, Sendai, Miyagi, Japan; Institute of Development, Aging and Cancer, Tohoku University, Sendai, Miyagi, Japan; Tohoku University, Sendai, Miyagi, Japan; Tohoku University, Sendai, Miyagi, Japan; Umeå University, Umeå, Vasterbottens Lan, Sweden; Umeå University, Umeå, Vasterbottens Lan, Sweden; Institute of Development, Aging and Cancer, Tohoku University, Sendai, Miyagi, Japan

## Abstract

**Background:**

While multiple modifiable lifestyle factors and disease management have been highlighted for preventing dementia and ameliorating neurodegeneration, women and the disadvantaged socioeconomic status (SES) population still bear disproportionate burdens. Objective: Investigate and compare the potential pathways of SES, lifestyle factors, imaging biomarkers, and cognition in two community dwelling cohorts in Japan and Sweden. Subjects: The Kumamoto Cohort included 576 cognitively healthy females (73.66 ± 5.96 years); the Betula Cohort included 195 cognitively healthy females (63.91 ± 13.41 years).

**Methods:**

We constructed structural equational modeling by lifestyle factors including exercise, social activity, sleep, drinking, and smoking status; disease conditions included obesity, diabetes, hypertension, and depressive disorder; brain imaging biomarkers included regional gay matter volume (GMV) and cortical thickness obtained from T1 weighted magnetic resonance imaging scans and global cognition score. We also examined SES-related gray matter volume and cortical thickness map locations at the whole brain level.

**Results:**

SES was positively associated with GMV of limbic lobe (not cortical thickness), Kumamoto Cohort: standardized direct β =0.21 (0.13;0.28); Betula Cohort: standardized direct β =0.27 (0.13; 0.41). This SES-GMV association was mediated by disease conditions and lifestyle in Kumamoto Cohort: indirect β =-0.013 (0.001; 0.054). We also found several regions, including the medial frontal gyrus, superior frontal gyrus, hippocampus, and thalamus, were commonly sensitive to SES status in two cohorts. Conclusions: Although the observational nature of the study precludes proof of causality, our findings suggest that promoting disease management is crucial to tackling the neurodegeneration burden in the female facing SES disparities.

